# Metacognition and Cognitive Flexibility in Autistic and Neurotypically‐Developing Populations

**DOI:** 10.1002/brb3.70668

**Published:** 2025-07-10

**Authors:** Mikhail Ordin, Natàlia Barbarroja, Leona Polyanskaya, Héctor M. Manrique, Miguel Castelo‐Branco

**Affiliations:** ^1^ Laboratory of Language, Metacognition and Decision‐Making Universidade de Coimbra Coimbra Portugal; ^2^ Department of Linguistics and Basque Studies, Centro de Investigación Micaela Portilla University of the Basque Country UPV/EHU Vitoria‐Gasteiz Spain; ^3^ Faculty of Psychology and Education Sciences University of Coimbra Coimbra Portugal; ^4^ Department of Psychology and Sociology Universidad de Zaragoza Aragon Spain; ^5^ Faculty of Medicine University of Coimbra Coimbra Portugal; ^6^ Coimbra Institute for Biomedical Imaging and Translational Research (CIBIT) Institute of Nuclear Sciences Applied to Health Coimbra Portugal

**Keywords:** cognitive flexibility, decision confidence, mental rotation, metacognition, visuospatial cognition

## Abstract

**Purpose:**

Whether and how metacognition is altered in individuals with autistic spectrum disorder (ASD) is intensely debated. Metacognitive deficit is claimed to be related to cognitive inflexibility, accounting for restrictive behaviors in ASD individuals. We wanted to test this hypothesis by measuring metacognition in ASD and in matched neurotypically developing (TD) control samples in a task that relies on visuo‐spatial cognition, in which ASD allegedly have an advantage.

**Methods:**

We measured metacognition in a 3D mental rotation task. Additionally, we administered a trading game: players had to figure out the rules for maximizing the profit on each transaction. These rules changed in the middle of the game, which required that players modify their strategy to keep the profit at maximum. We measured both learning efficiency (how fast players extract the rules) and re‐learning speed (cognitive flexibility, how fast learners could adjust their behavioral responses after rules are changed).

**Results:**

TD outperform ASD individuals in terms of accuracy in mental rotation but exhibited lower metacognitive efficiency (i.e., were less aware when they were more likely to make an error). No differences in learning efficiency and cognitive flexibility between TD and ASD individuals were observed. Neither did we observe an association between cognitive flexibility and metacognition. Nevertheless, both in ASD and TD populations, overconfidence in one's decisions is negatively correlated with cognitive flexibility, but not with learning efficiency.

**Conclusion:**

ASD individuals can have superior metacognition in tasks that rely on visuo‐spatial cognition. Cognitive flexibility is diminished by overconfidence, not by metacognitive deficit.

## Graphical abstract

ASD individuals can have superior metacognition in the tasks that rely on visuo‐spatial cognition. Cognitive flexibility is diminished by overconfidence, not by metacognitive deficit.

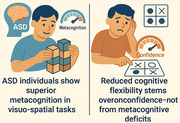

## 1 Introduction

Metacognition is the ability to track one's own cognitive processes and mental states, evaluate decisions and uncertainty associated with such decisions and the environment in which decisions are made, and control one's behavior to optimize it to the environment and decision consequences (Flavell 1979; Nelson 1996; Kepecs et al. 2008). Under laboratory conditions, metacognition is often studied by means of retrospective confidence when participants need to assign a confidence rating to their decisions on each experimental trial. Metacognition allows the detection of cases when an error is more likely and assigning lower confidence ratings to such decisions. Enhanced metacognition is reflected in confidence ratings better discriminating between correct and wrong responses (Maniscalco and Lau 2012). Hence, metacognitive measures often rely on how closely confidence ratings can track accuracy. This is how we operationalize metacognition in this study. Metacognition is thought to be altered in individuals with ASD, although the evidence is inconclusive. Some studies even show enhanced metacognitive abilities in ASD individuals. The variability in metacognitive competence can potentially account for the variability in the most distinctive phenotypic characteristics of ASD individuals, such as impairments in social interaction and communication (Corbett et al. 2009; Rodgers et al. 2017; Sanders et al. 2008), restricted interests, and stereotyped behavior (American Psychiatry Association 2013). We wanted to test the association between cognitive flexibility and metacognition in a task that relies on visuo‐spatial cognition, in which individuals with ASD allegedly have an advantage over TD individuals.

### 1.1 ASD and Metacognition

After Frith and Happe (1999) argued in their seminal paper that impaired theory of mind (ToM) abilities of ASD (autistic spectrum disorder) individuals lead to impaired metacognitive abilities, a new plethora of studies was published, either supporting or challenging this claim (see van der Plas, Mason, and Happé 2023, and Carpenter and Williams 2023 for the most recent reviews and meta‐analyses). Despite the general consensus that ASD individuals have difficulties in ToM‐based tasks, the topic of whether ASD individuals have altered metacognition is still unresolved and hotly debated in the literature, with new studies often yielding contradictory results. A relatively recent meta‐analysis of secondary empirical data revealed a very moderate reduction in metacognitive accuracy in ASD population of children, yet an almost negligible difference between autistic and non‐autistic adults (Carpenter and Williams 2023), which also corresponds to primary empirical data by Grainger et al. 2014; 2016a; 2016b). Importantly, the effect is varied depending on the perceptual modality (e.g., visual or auditory), operational domain (e.g., reasoning, visuospatial perception, memory, language), task difficulty, and measures of metacognition (feeling of knowing, judgment of learning, judgment of performance, judgment of confidence) and how metacognitive skills are quantified (e.g., gamma correlations, M‐ratio, difference between predicted and actual performance, and difference in confidence ratings on correct versus incorrect responses). The nature of the task might also play a role. Carpenter et al. (2019), Grainger et al. 2014; 2016a; 2016b) and Nicholson et al. 2019; 2021) reported that children and even adults with ASD have lower metacognitive accuracy if metacognition is measured in an explicit way (when the instructions are provided explicitly and people need to consciously evaluate their own performance, either verbally or by assigning a confidence rating of how sure one is in their own response), but such differences disappear once the measures of metacognition are inferred from the behavior of the participants (implicit measures such as reaction time—how long the decision process is taking, or number of false starts—how often the participant changes his/her mind before making a final decision).

Moreover, ASD individuals represent a very heterogeneous population, and samples of ASD individuals are not always comparable across studies. Researchers use different tools to diagnose ASD, from standardized diagnostic tests such as the ADOS or ADI‐R (clinically approved and properly applied only by trained clinicians) to self‐assessment questionnaires. The use of different diagnostic tools makes it even more difficult to compare the results across studies and may contribute to controversies. Another important fact, often neglected, is the developmental age and how it impinges on different sensory, attentional aspects and behavioral responses. For instance, the percentage of time that infants spend looking at lips as compared to the eyes in autistic babies is not different from that in TD‐infants (Jones and Klin 2013); however, later in childhood, ASD kids devote more time to attending to lips than TD children—in other words, they do not shift attention from lips to eyes in the course of development (Klin et al. 2002; Jones et al. 2008). Also, in infants with ASD, visual sensory information is subdued in favor of interoceptive cues (Schauder et al. 2015), yet this again changes, when they reach late childhood when they take in visual information similar to TD. These differences in what ASD individuals attend to at different developmental stages and greater variability—compared with TD individuals—in the rate at which different developmental stages are reached can partially explain the mixed results across studies.

Moreover, the autistic spectrum may extend to the TD population (Geurts et al. 2013; Nummenmaa et al. 2012). Questionnaires like RAADS‐14 (14 statements) or Autistic Quotient (AQ) questionnaire (50 statements, which participants need to support on a 4‐point Lickert scale, Baron‐Cohen et al. 2001) were designed to detect autistic traits in people who are either not diagnosed with clinical tools and/or who meet the criteria for pervasive development disorder yet struggle with social interaction, mindreading, repetitive stereotypical behaviour and restrictive interests. The strength to which autistic traits are expressed in a single individual is referred to as AQ. Even though the AQ is a standardized measure that can be applied to ASD and TD individuals, the data concerning whether the AQ and metacognitive ability are related are not conclusive either. Moreover, researchers sometimes report inconsistent results when the same task is administered to clinically diagnosed individuals and TD individuals with high AQs. Williams et al. (2018), for example, argued that there is no relationship between the AQ score and metacognition; however, diagnosed ASD kids had lower metacognition compared to not clinically diagnosed kids with high AQ. In contrast, Carpenter et al. (2019) reported no differences in metacognition between the diagnosed and non‐diagnosed groups but reported a strong correlation between the AQ and metacognition. Van der Plas used a shorter questionnaire (RAADS‐14) to measure autistic traits in a TD sample and reported that metacognitive accuracy is negatively correlated with autistic traits, referring to impairments in social interaction and communication. Moreover, other phenotypic characteristics related to autistic spectrum disorder—restricted interests and stereotyped behavior—are not correlated with metacognitive abilities. It was also shown that metacognitive skills were diminished in individuals with diagnosed ASD compared with TD individuals with high AQ, who were matched for age, sex, IQ, and educational level. Embon et al. (2023) used the AQ test by Baron‐Cohen et al. (2001) and reported that in the TD population, the AQ is not correlated with metacognitive skills, at least in a low‐level visual perception task. The association was not found on the AQ subscale pertaining to social skills, which contradicts the findings of van der Plas et al. (2021), despite similarities in tasks and measures of metacognition, and may be linked to different AQ assessment tools (AQ quotient test designed by Baron‐Cohen et al. 2001 vs. RAADS‐14). This overview also suggests that the results obtained for TD individuals with high AQs might nevertheless be not generalizable to a diagnosed ASD population and might not be generalizable to individuals evaluated by different diagnostic tools.

### 1.2 ASD and Mental Rotation

Several studies even showed enhanced visuospatial cognition in the adult ASD population (Falter et al. 2008; Joseph et al. 2009; Mottron et al. 1999; O'Riordan 2004; Soulieres et al. 2011), especially in low‐level visual perception (Caron et al. 2006; Happé and Frith 2006; Vulchanova et al. 2012). Moreover, visuospatial cognition is spared in ASD individuals even when they exhibit deficits in executive functions, attention, language skills, memory, learning and social cognition (Narzisi et al., 2013). Some theories link attention to details and enhanced visual perception with the socio‐cognitive deficits observed in diagnosed individuals (Dakin and Frith 2005; Mottron et al. 2006; Pellicano and Burr 2012; Plaisted et al. 1998). Attention to details and recognition of minute differences in visual scenes might suggest that visual information is subject to a higher degree of conscious processing in ASD individuals, and this might result in more efficient explicit metacognitive monitoring. Empirical data from the studies outlined above do not align well with this theory‐based suggestion. Even though empirical studies are inconsistent, they either show metacognitive deficits in ASD populations compared with TD populations or do not reveal significant differences between the groups. This gap between empirical data and what could be observed on the basis of theoretical implications could be accounted for by the nature of the visual tasks presented and by the participants’ age (infants and children direct attention inwards and neglect visual external information more often than adolescents and adults do). In earlier studies, differences in metacognition between ASD and TD populations were explored in low‐level perception tasks (e.g., visual discrimination, visual search, and identification; see Narzisi et al., 2013 and Simmons et al., 2009 for review), whereas explicit metacognition might be more engaged by higher‐level cognitive processes that rely on visual perception. Therefore, we compared the metacognitive efficiency of ASD and TD individuals in a mental rotation task (Shepard and Metzler 1971).

Mental rotation is clearly differentiated from other tasks engaging visuospatial cognition. ASD individuals usually do not exhibit complications with tasks that require object rotation, yet they have severe problems using their body for reference to perform spatial transformations (Muth et al. 2014; Pearson et al. 2015). Therefore, this task allowed for testing several important hypotheses related to autism and made this task one of the most used paradigms to explore visuospatial performance in ASD individuals. Sex differences are typically reported in mental rotation tasks, with males, as a rule, performing better than females (Aleman et al. 2004; Astur et al. 2004; Brosnan et al. 2010; Knickmeyer et al. 2008; Linn and Petersen 1985; Tapley and Bryden 1977; Voyer et al. 1995; for debate review see Falter et al. 2008), and this task was put to use to test the “extreme male brain hypothesis” (Baron‐Cohen 2001) or theories related to linking enhanced visuospatial cognition and central disturbances of autism (Dakin and Frith 2005; Mottron et al. 2006; Pellicano and Burr 2012; Plaisted et al. 1998). However, while many studies indeed reported higher performance in the mental rotation task by ASD individuals (Falter et al. 2008; Soulieres et al. 2011), many recent studies failed to replicate this finding (Rohde et al. 2018; Larson et al. 2024).

Heterogeneous results might be explained by more versatile strategies (some might be sex‐determined; Larson et al. 2024) in the ASD than in the TD population. ASD individuals use neural resources differently (Damarla et al. 2010; Larson et al. 2021; Muth et al. 2014) and have more low‐level visual information for decision‐making in a high‐level visual cognitive task like mental rotation (Caron et al. 2006; Happé and Frith 2006; Vulchanova et al. 2012). Engagement of different cognitive strategies, which must be constantly monitored, might enhance metacognitive sensitivity. Additionally, a larger amount of low‐level visual information might provide more data for post‐decision evaluation of one's responses in a high‐level cognitive task, when the responses are slower than in low‐level tasks, and low‐level information might be processed for a longer time. This potentially further enhances metacognition in the mental rotation task in the ASD population (even if the cognitive performance in the task per se is on par with that demonstrated by the TD controls). In our study, we will test the prediction of more efficiency metacognition in a mental rotation task by an ASD compared to a matched TD population.

### 1.3 ASD and Cognitive Flexibility

Although autism and its varieties (Kanner's syndrome, Asperger's disorder, childhood disintegrative disorder, or Rett syndrome) are no longer considered distinct diagnoses, phenotypical manifestations such as deficits in social communication and interaction, difficulties with social‒emotional reciprocity, and restricted and repetitive patterns of interest and behaviors are considered stereotypical symptoms of autistic spectrum disorder (APA; Corbett et al. 2009; Rodgers et al. 2017; Sanders et al. 2008). Repetitive behavior in individuals with ASD is often observed not only at the motor level but also at the level of decision‐making and diminished cognitive flexibility (Fujino et al. 2019; Landry and Mitchell 2021; Geurts, Corbett, and Solomon 2009; Fujino et al. 2017a; Fujino et al. 2017b; Van Eylen et al. 2011). Cognitive flexibility is the ability to switch between decision strategies or mental processes in order to generate the most appropriate behavioral response given one's state of mind and the conditions of the environment (Champagne‐Lavau et al. 2012; Tei et al. 2018a). Compromised cognitive flexibility does not allow selective and deliberate switching between tasks or different sets of rules, hence inhibiting the ability to adapt to changes in the environment or to situations in which changes in regularities require modifying decision strategies for optimization of the results (Armbruster et al. 2012; Scott 1962). A whole body of works on autism has led some researchers to suggest that important differences in decision‐making strategies and cognitive flexibility between ASD and TD individuals can be attributed to altered (explicit) metacognition in the former (Carruthers 2009a; 2009b; Frith and Happé 1994; Happé and Frith 2006; Van Eylen et al. 2011; Nicholson et al. 2019). This suggestion has not yet been fully tested empirically. Previous research has focused either on cognitive flexibility and decision‐making or on metacognition (which is often investigated in conjunction with ToM capabilities) in individuals with ASD. In this study, we wanted to explore whether metacognition is indeed associated with cognitive flexibility in ASD and TD individuals.

Van Eylen et al. (2011) reported that ASD individuals exhibit cognitive flexibility deficits only in those paradigms in which people have to extract the rules themselves, especially when people have to detect cases where a new strategy should be developed or applied to optimize their behavioral response. When the role of explicit instruction increases, the deficit in cognitive flexibility in ASD individuals decreases. This suggests that ASD individuals can indeed acquire and effectively apply different strategies, yet they have problems detecting when a particular strategy is no longer optimal. When they are explicitly informed about the need to change strategies, an optimal response is given, indicating a problem with flexible implementation of what is learned rather than with learning per se. As ASD individuals do not exhibit impoverished cognitive flexibility when explicit instructions are given (Van Eylen et al. 2011), in this study we designed an experimental paradigm that required participants to detect independently, based on evaluation of their behavioral responses, when a previously developed strategy no longer led to an optimal response. Given that this implicit nature of the task required self‐monitoring of one's performance, we hypothesized that it might be affected by general metacognitive monitoring.

Alternatively, cognitive inflexibility in ASD individuals might be related to lower decisiveness and the need to accumulate more evidence that an exploited strategy or behavioral pattern is no longer optimal before other options are explored. The decisiveness score tends to be lower in ASD individuals than in TD individuals (Fujino et al. 2019; Brosnan et al. 2014), which should be reflected in lower metacognitive bias in ASD individuals. In this study, we also wanted to test whether diminished cognitive flexibility is related to metacognition or simply to lower overall confidence (under‐confidence) of individuals, and whether cognitive flexibility in autism is indeed atypical, or it is modulated by metacognition or overall confidence in both TD and ASD populations.

## 2 Method

The experimental protocol and recruitment procedures were approved by the ethical committee of the University of the Basque Country (EHU/UPV, approval record M10_2022_146MR2) for data acquisition in Spain, by the ethical committee of the Psychology Faculty of the University of Coimbra (approval number CEDI/FPCEUC:70/0PE) for neurotypically developing participants, and by the ethical committee of the Medical Faculty of the University of Coimbra (approval number CEDI/FMUC:CE‐145/202) for autistic participants.

### 2.1 Participants

We recruited 33 individuals who were diagnosed by the ADOS‐2 at different centers in central Portugal (Viseu and Coimbra) and Spain (Sabadell). Individuals in Sabadell were Spanish‐Catalan bilinguals (*N* = 18, 2 females) and acquired both languages from birth. Individuals in Portugal (*N* = 15, 3 females) could hold a conversation in English language on routine daily topics, but they acquired a second language later in life through the school system. The ASD sample is male‐dominant, which corresponds to the gender distribution in the population of individuals who are formally diagnosed with autistic spectrum disorders (80% males, Werling and Geschwind 2013). Although there is a debate as to whether females are better imitators and more often go undiagnosed because they can imitate the behavioral patterns of typically developing individuals, or whether autistic spectrum disorders are indeed prevalent in males, we are not discussing this topic because it falls outside the scope of our work. The recruited participants were not cognitively impaired; they passed the IQ threshold (set to 90, Wechsler or Kaufman test) to be considered highly functional autistic individuals. The participants varied in age from 16 to 35 years old (*M* = 21), with no difference between the Spanish and Portuguese samples.

The sample size of ASD individuals was determined by the number of individuals diagnosed with ASD who volunteered to participate in the study. As a control sample, we recruited TD individuals in the same geographic areas, matched for age, sex, and linguistic background with ASD individuals (once the ASD sample was collected, we recruited the same number of TD individuals by finding a sex‒age pair for each ASD individual). In Spain, all participants received remuneration of 10 euros. In Portugal, participants were volunteers, and according to national law, monetary compensation was prohibited.

We ran a sensitivity power analysis to estimate the effect size that could be detected with this sample size via ANOVA with two factors (region–2 levels and groups—ASD vs. TD). Although region was not a factor of interest, we cannot exclude the possibility of significant differences in metacognition and cognitive flexibility between countries, especially considering the fact that participants in Spain were bilingual, and bilingualism is known to enhance metacognition in high‐level cognitive tasks (Ordin et al. 2020; Polyanskaya et al. 2022). Moreover, the presence of monetary compensation in only one group might also result in between‐region differences. The analysis revealed that, if differences between groups and regions indeed exist, the difference will be significant at alpha = 0.05 if the minimal effect is *f* = 0.35 (η^2^_p_ = .06), given equal variance within each group (it was indeed confirmed before running the main analysis) and the independence of samples (the samples were collected by different researchers and participants falling into different groups did not know each other, meaning that this assumption is also complied with). The outcome of the analysis shows that with the acquired sample size, we would be able to detect an effect of any of the factors (group or region) if it accounts for at least 6% of the variance in the difference between factor levels. Given these results, we assume that with the acquired sample size, we achieve adequate statistical power.

The duration of the experiment was approximately one hour, and the study was performed individually with each participant.

### 2.2 Procedure and Stimuli

**FIGURE 1:**
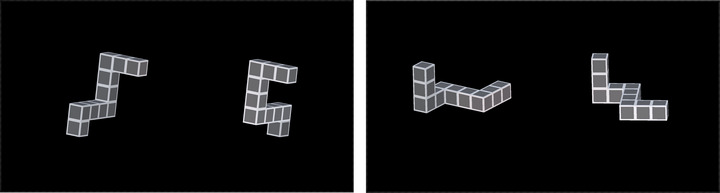
Stimuli example. On the left panel, the shapes are different, and no mental rotation can result in alignment of these shapes. On the right panel, the shapes are identical, and alignment is possible by 60 degrees yawing (rotating the second shape along the y‐axis by 60 degrees).

Participants performed a mental rotation task, on which metacognitive ability was evaluated, and played a trading game to obtain a proxy measure of cognitive flexibility. All tests were administered in the native language of the participant. For mental rotation, we used 3‐D abstract shapes, each composed of 10 square blocks, as stimuli (see Figure 1). On each trial, participants saw two shapes, and they had to decide if they were identical (and can be aligned when the second shape is rotated around X or Y axis) or not. In 36 trials, the shapes were different,;in 18 trials, the shapes could be aligned by rotating around the X axis; and in 18 trials, alignment was possible by mental rotation around the Y axis (72 trials in total). The rotation varied from zero to 340 degrees, with 20‐degree steps. On each trial, participants had to use a four‐point scale to indicate how sure they were in their response whether the presented shapes were identical or not. The order of trials was randomized for each participant. The responses were registered using a mouse.

In a trading game, participants were informed that they would have to sell four minerals to three alien species. As different species have different needs, a mineral that is valuable for one alien might be less valuable for another, hence different aliens will pay different amounts for different minerals. On each transaction, a participant could get either 5 or 10 or 20 coins. On each trial, participants saw an image of the mineral in the upper left part of the screen, and three aliens (of equal size) in the bottom half of the screen, marked by “1”, “2,” and “3” symbols, from left to right. Participants had to press the “1”, “2” or “3” button on the keyboard to indicate whom of the aliens they wanted to sell the displayed mineral. Once the decision was made, participants learned how many coins they earned on the transaction, and the next trial started. In the upper right part of the screen, the number showed the total amount earned for all previous transactions. Participants were informed that their task was to maximize the gain (total amount earned).

The matrix of the award amount associated with each alien‐mineral pair is given in Table 1. Mineral 4 was equally valuable for each species. The rationale for including this mineral was to find out whether participants had individual preferences for trading with a particular alien (to whom they would sell mineral 4), and to consider this preference in subsequent analysis. However, preferences for trading with a specific alien when the reward did not vary were not observed.

**TABLE 1:** The matrix of income associated with selling minerals to aliens.

	Mineral 1	Mineral 2	Mineral 3	Mineral 4
Alien 1	5	10	20	10
Alien 2	20	5	10	10
**Alien 3**	10	20	5	10

First, each mineral was presented 12 times. The order in which minerals were presented was randomized, and then kept constant across all participants. After 48 trials, the rules were reversed: the alien‐mineral combinations that yielded 5 coins in the first 48 trials started producing 20 coins; the pairings that yielded 20 coins started giving 5 coins of income. After the reversal, each mineral was presented 5 times in random order that was kept constant across all participants. In total, the experiment had 68 trials.

### 2.3 Analysis

The mental rotation task was administered to evaluate metacognitive ability rather than visuospatial ability. As metacognition is a correspondence between correctness and confidence (how closely confidence tracks accuracy), we only used accuracy in the mental rotation and ignored reaction time, which is frequently analyzed when performance in mental rotation per se and visuospatial ability represent the key interest of the researchers.

Explicit metacognition is manifested by higher confidence ratings assigned to correct than to wrong responses. We first calculated the average confidence per participant, separately on correct and wrong responses, and ran repeated‐measures ANOVA with accuracy (correct vs. incorrect) as the within‐subject factor, group (ASD vs. TD) and region (Catalan vs. Portuguese) as between‐subject factor. A significant effect of accuracy would signal that explicit metacognition is engaged by the task, and would provide justification for comparing metacognition across ASD and TD samples.

To compare metacognitive skills between ASD and TD groups, we used a signal detection theory (SDT) approach (Galvin et al. 2003; Fleming and Lau 2014; Maniscalco and Lau 2012; 2014). For cognitive performance, correct responses were labeled either as hits (two identical shapes are identified as identical) or correct rejections (two different shapes are identified as different; i.e., not identical), and incorrect responses were labeled either as misses (when participants miss the fact that two presented shapes are identical and respond that they are different) or false alarms (two different shapes that cannot be aligned by rotating are identified as identical). At the metacognitive level, correct responses, to which participants assign high confidence, are conceptualized as meta‐hits, and correct responses, to which low confidence is assigned, are conceptualized as meta‐misses. Incorrect responses, to which high confidence is assigned, are treated as meta‐false alarms, and incorrect responses, to which low confidence is assigned, are considered meta‐correct rejections. Using these conceptualizations, metacognitive sensitivity of each individual was estimated as the ability of the confidence rating to discriminate between correct and incorrect responses. The measure of metacognitive sensitivity is meta‐d' (an estimated d', which would be observed if confidence ratings could discriminate between correct and wrong responses optimally, or a pseudo‐d' that would perfectly fit confidence ratings). Meta‐d' is a measure of how sensitive a given individual is to his own mental states when reporting confidence in one's decision. Although this measure is not affected by the metacognitive bias (the individual over‐ or under‐confidence, manifested as a tendency to assign higher or lower confidence ratings overall), it scales with cognitive performance. Typically, an individual with a higher performance in the task also has higher meta‐d' compared to an individual with lower performance because the former exhibits higher d', i.e., is more sensitive to the signal, which is used to make confidence judgments. To control for idiosyncratic or group differences in task performance, metacognitive efficiency is calculated as the M‐ratio (meta‐d'/d'), which is a measure of individual explicit metacognitive abilities given a particular performance level of this individual. In other words, metacognitive sensitivity (meta‐d') is the sensitivity the amount of signal that is provided by the cognitive system for metacognitive judgment (sensitivity to that part of the signal, which is subject to metacognitive judgment). Metacognitive efficiency is how effectively an individual can operate with the signal that his cognitive system makes available for metacognitive judgments. M‐ratio allows for comparing individuals and groups that differ in the task performance, and to compare across tasks with different levels of difficulty.

We calculated metacognitive bias for each participant as the averaged confidence on all responses, irrespective of whether they are correct or incorrect, which allowed comparing over/under‐confidence between groups. For estimating meta‐d', we adopted a hierarchical Bayesian estimation proposed by Flemming (2017), because, unlike maximum likelihood estimation (MLE) models, this approach is advantageous given a limited number of trials, does not require data padding (e.g., adding a small value to empty cells, if an individual did not give any correct or wrong responses with a particular level of confidence, or when the number of responses with particular levels of confidence is substantially lower, which can happen if an individual prefers to assign extreme confidence values or intermediate confidence values on the confidence scale). Although MLE methods are superior in low‐level perception or psychophysics experiments with hundreds of trials, we assume that a hierarchical Bayesian estimation is more robust in higher‐level cognitive tasks when the number of trials is limited by practical reasons.

The trading game was aimed to assess cognitive flexibility. For the analysis of the data from the trading game, we calculated how many trials each participant needed to learn the optimal alien‐mineral pairing to maximize the gains. Learning was considered to be completed once two conditions were met: (1) a participant achieved maximal award on three consecutive transactions when it was possible to earn 20 coins (i.e., transactions when mineral 4 was traded and hence earning 20 coins was not possible were not considered interrupting the sequence of consecutive transactions with maximal award); and (2) the performance remained stable and did not drop to two consecutive transactions with minimum award (i.e., 5 coins) afterwards. The number of trials needed to learn the optimal alien‐mineral pairing was a proxy for individual learning speed. We then estimated the number of trials needed to relearn the rules once rule reversal occurred. Relearning was considered successful once the same conditions outlined above were met. We used individual relearning speed as a measure of cognitive flexibility.

## 3 Results

### 3.1 Mental Rotation (Metacognitive Efficiency)

**FIGURE 2:**
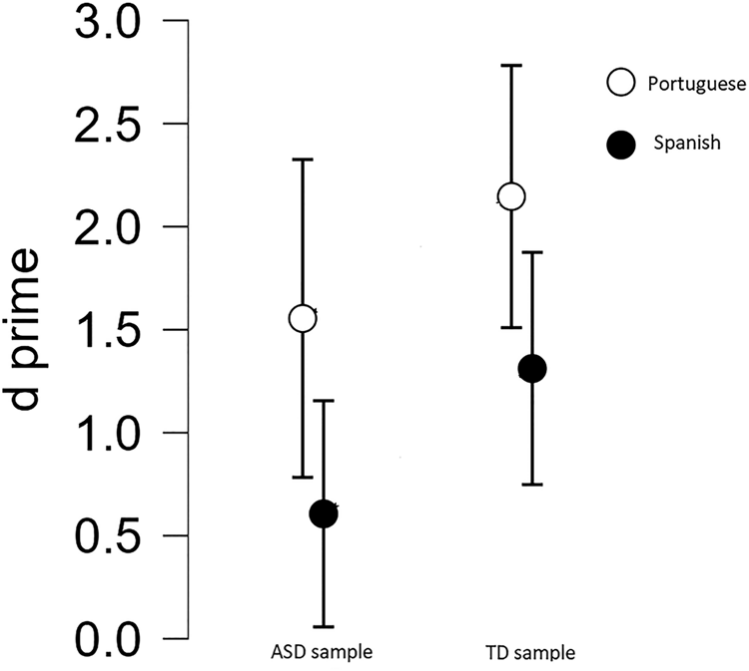
Cognitive performance of participants in the mental rotation task. Error bars display ± 95%CI.

An ANOVA with group (TD vs ASD) and region (Spain vs Portugal) as factors and *d*' as the dependent variable revealed a significant effect of group, *F* (1, 62) = 4.84, p = 0.032, η_p_^2^ = 0.072, and region, *F* (1, 62) = 9.143, *p* = .004, η_p_^2^ = 0.129, with no significant interaction, between the factors, *F* (1, 62) = 0.037, *p* = 0.848, η_p_^2^ = 0.000. (The same analysis with the number of correct responses as a dependent variable yields the same result pattern, with a stronger effect). TD participants gave more correct responses than ASD participants did, and Portuguese participants gave more correct responses than Spanish participants did. This resulting pattern is shown in Figure 2.

**FIGURE 3:**
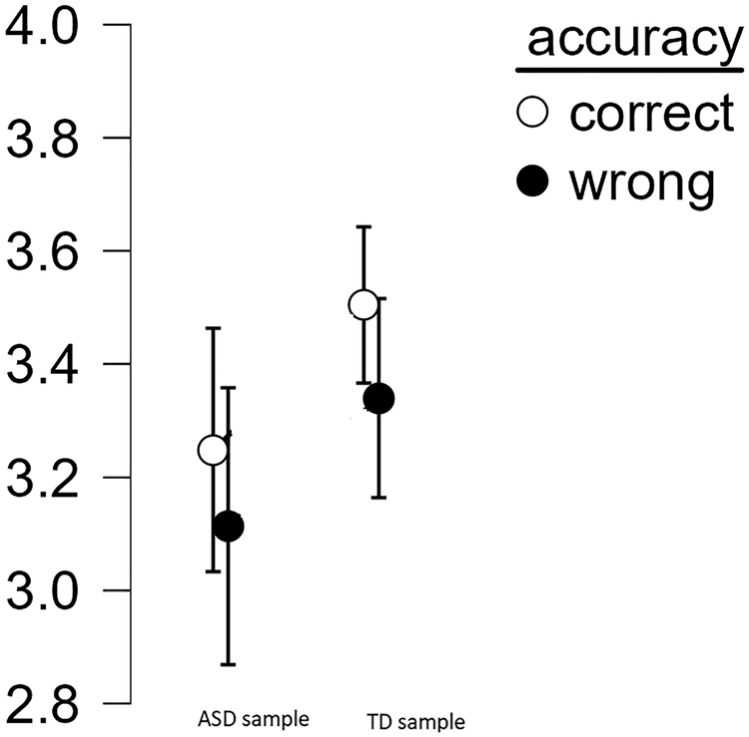
Mean confidence on correct and wrong responses given by autistic and typically developing individuals. Error bars display ± 95%CI.

To ensure that metacognition was at all engaged by the task, we compared confidence ratings assigned to correct and incorrect responses via a repeated‐measures ANOVA with accuracy (correct vs. incorrect) as a within‐subject factor, group (TD vs. ASD), and region (Spain vs. Portugal) as between‐subject factors, and the confidence rating as a dependent variable. The analysis revealed a significant effect of accuracy, *F* (1, 60) = 20.112, *p* < 0.001, η_p_^2^ = 0.251. Confidence was overall higher on correct than on incorrect responses, which means signals that the metacognitive system was engaged in all samples (Figure 3). The interaction of accuracy with any other factor was not significant: for the interaction with group, *F*(1, 60) = 0.112, *p* = 0.728, η_p_^2^ = 0.002; for region, *F* (1, 60) = 2.68, *p* = 0.107, η_p_^2^ = 0.043. The effects of group, *F* (1, 60) = 2.942, *p* = 0.091, η_p_^2^ = 0.047; region, *F* (1, 60) = 1.426, *p* = 0.237, η_p_^2^ = 0.023; and the group*region interaction, *F* (1, 60) = 0.545, *p* = 0.463, η_p_^2^ = 0.009, are not significant. This means that metacognitive bias is not different across samples (i.e., there was no trend showing that individuals in any sample exhibit over /under‐confidence compared with individuals in other samples). The three‐way interaction between accuracy, region and group was not significant either, *F* (1,60) = 0.464, *p* = 0.499, η_p_^2^ = 0.008.

**FIGURE 4:**
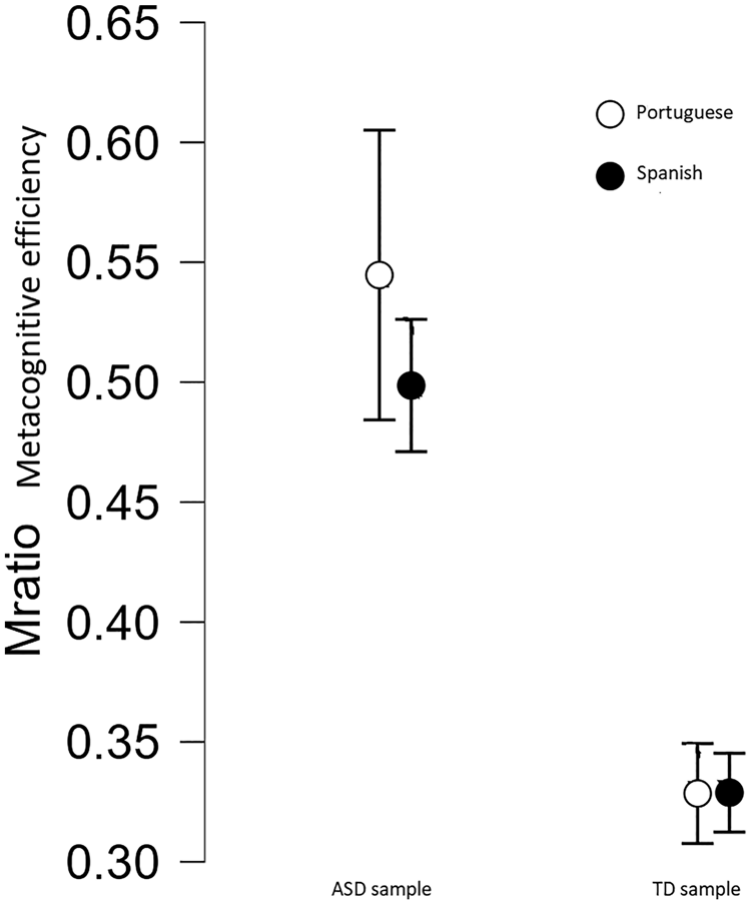
Metacognitive sensitivity (measured as M‐ratio) in autistic and typically developing individuals. Error bars display ± 95%CI.

To compare metacognitive efficiency (M‐ratio) between TD and ASD individuals, we ran an ANOVA with group (TD vs. ASD) and region (Spain vs. Portugal) as factors. The analysis showed that M‐ratio was affected only by the group, *F*(1, 62) = 146.813, *p* < 0.001, η_p_^2^ = 0.703, with neither regions, *F*(1, 62) = 2.057, *p* = 0.157, η_p_^2^ = 0.032, nor group × region interaction, *F* (1, 62) = 2.121, *p* = 0.15, η_p_^2^ = 0.033 reaching the significance threshold. Intriguingly, metacognitive efficiency in the mental rotation task was substantially higher in ASD compared to TD individuals. This pattern is shown in Figure 4.

As we are interested in differences between the ASD and TD populations in metacognitive efficiency and we want to be more certain that such differences are generalizable across other geographical regions (not only Spain and Portugal), we ran mixed regression models with group as a fixed factor and region as a random factor (which implies that there might be other regions that are not included in the analyzed samples). The effect of the group was significant, *t* = 8.347, *p* = 0.014, *B* = 0.096 (intercept for the group was also significant, *t* = 36.933, *p* < 0.001, *B* = 0.425; the contrast estimate was also significant, *B* = 0.192, *p* < 0.001). As the results of the mixed model with region as a random factor and the ANOVA analysis converged, we can be more certain of the generalizability of the differences between the ASD and TD populations across populations in other regions.

### 3.2 Trading Game (Cognitive Flexibility)

We used re‐learning speed (how fast individuals re‐learn the new rule after the matches between the reward amount and the mineral are reshuffled across trading aliens). Re‐learning speed may be modulated by learning speed, which is supported by a strong and significant correlation (Spearman's) between them, *ρ* = 0.363, *p* = 0.003: faster learning is associated with faster re‐learning. For the analysis, we designed an AN(C)OVA model with group and region as factors and added learning speed as a covariate to regress out its influence on re‐learning. The data did not show any significant differences between ASD and TD individuals, *F*(1, 61) = 0.94, *p* = 0.336, η_p_^2^ = 0.015; or between individuals from Portugal or Spain, *F*(1, 61) = 0.751, *p* = 0.389, η_p_^2^ = 0.012. The interaction between factors was not significant either, *F*(1, 61) = 0.007, *p* = 0.931, η_p_^2^ = 0.000. As expected from the reported correlations, the effect of learning speed was significant, *F*(1, 61) = 9.49, *p* = 0.003, η_p_^2^ = 0.135.

### 3.3 Association Between Cognitive Flexibility, Metacognitive Efficiency and Metacognitive Bias (Over/Under‐Confidence)

To probe for the association between metacognitive skills and cognitive flexibility, we estimated Spearman's correlation coefficients between M‐ratio and re‐learning speed, separately in the ASD and in TD samples. We observed significant correlations neither in the ASD, *ρ* = 0.156, *p* = 0.386, *z* = 0.157, nor in the TD samples, *ρ* = 0.159, *p* = 0.377, *z* = 0.16 (the difference between correlation strength is not significant either).

However, we observed a significant correlation between learning speed and M‐ratio scores in the ASD group, *ρ* = 0.384, *p* = 0.027, *z* = 0.405; yet in the TD group this correlation was not significant, *ρ* = 0.033, *p* = 0.854, *z* = 0.033; and the difference in the strength of correlation between the groups is not significant either, Z_observed_ = 1.44, *p* = 0.075. These results suggest that ASD individuals, unlike TD participants, benefit from enhanced metacognitive monitoring—even though we measured metacognition in a different operational domain from the one in which we measured learning speed. Enhanced self‐awareness is manifested across domains and allows for better estimation of consequences of previous decisions and better integration of these consequences when planning the next move in the trading game to maximize profit. However, the conclusion is tentative, given that the difference in correlation strengths between learning and metacognitive efficiency in TD and ASD populations is not significant.

Further on, we verified that the metacognitive efficiency is indeed independent of the metacognitive bias in the same task (*r* = 0.107, *p* = 0.55 for TD and *r* = 0.107, *p* = 0.554 for ASD populations), and whether meta‐bias interferes with cognitive flexibility (re‐learning speed) and learning efficiency (learning speed). For the latter, we calculated nonparametric Spearman correlations between the overall confidence of each individual and learning speed, *ρ* = –0.198, *p* = 0.27 for the TD population, and *ρ* = ‐0.205, *p* = 0.252 for the ASD population. The correlations were negative, suggesting that over‐confidence negatively impact learning efficiency, but the result is not significant.

However, Spearman correlations between re‐learning (once the rules were changed) and overconfidence were significantly negative, *ρ* = –0.395, *p* = 0.023, *z* = –0.417 for the TD and *ρ* = –0.583, *p* < 0.001, z = –0.667 for the ASD populations, with no significant difference in the correlation strength between the groups, Z_observed_ = 0.968.2, *p* = 0.166. Considering that faster learners with an equivalent level of cognitive flexibility may learn a new set of rules faster, we verified this result pattern by calculating partial Spearman correlations between re‐learning speed and overall individual confidence, controlling for learning efficiency (learning speed). The result pattern still persists and survives the significance test both for the TD, *ρ* = 0.36, *p* = 0.04, *z* = 0.37, and for the ASD populations, *ρ* = ‐0.463, *p* = 0.008, *z* = 0.502, with no significant difference in the correlation strength between the groups, Z_observed_ = 0.481, *p* = 0.315. This pattern suggests that the tendency towards overconfidence negatively impacts cognitive flexibility, but not learning efficiency. Once an individual has learnt a set of rules and has successfully applied this knowledge, (s)he finds it harder to adapt and change the strategy by learning a new set of rules after the environment changes, and old set of rules no longer leads to optimal behavior. The average individual confidence at the group level being equal to 3.5 in both the TD and ASD samples, with confidence ratings ranging from one (minimum) to four (maximum), with a mean value of 2.5. None of the participants exhibited a mean individual confidence score less than 2.5. Therefore, we interpret the results in terms of overconfidence rather than under‐confidence: we do not say that under‐confidence positively impacts cognitive flexibility, but rather that overconfidence negatively impacts cognitive flexibility.

## 4 Discussion

The results showed that the ASD individuals are less accurate in the mental rotation task, but exhibited higher metacognitive efficiency in this task, compared to the TD individuals. No differences in cognitive flexibility were observed between the ASD and TD populations. Probably, it might be accounted by domain‐specificity of the metacognition (metacognitive efficiency in a specific task—mental rotation—has no effect on performance in a different, unrelated task that relies on different cognitive mechanisms and requires monitoring different cognitive strategies). Overconfidence in one's own decisions negatively impacts cognitive flexibility and compromises adapting to the environment once the regulations in the environment change and the previous strategies, developed based on the outdated regulations, no longer leads to optimal decisions. Overconfidence and learning, however, are not correlated. This pattern was observed in both groups, with no observable differences between the ASD and TD populations.

Many previous studies reported a metacognitive deficit in ASD population, whereas our work is in line with those studies that reported enhanced metacognition in ASD individuals in tasks that rely on the evaluation of sensory information and awards rather than prior explicit knowledge (e.g., Fazioli et al. 2024; Fazioli et al. 2025). One of the reasons for the contradictory results in the previous literature is related to variability in defining ASD samples (either on the basis of AQ and autistic traits, or formal diagnoses, or self‐reports and attending support centers for autistic individuals; mixing highly functioning individuals with average or above‐average IQ with those who require constant support from care givers due to severe impairments that make independent functioning in society impossible; etc.). In this study, we used strict criteria to define the population of interest: we focused on highly‐functioning individuals (able to sustain independence without constant supervision of care‐givers, but receiving regular support from specialists in support centers), formally diagnosed by the ADOS, with IQ scores showing average IQ level or above. An interesting question is why individuals in such a population have enhanced metacognition in a mental rotation compared with typically developing controls.

Metacognition, to a large extent, relies on the evaluation and monitoring of one's own mental states (because changes in mental state signal the likelihood of making an error at a decision time, or estimation of the likelihood of receiving an award, to which ASD individuals are very sensitive) (Fazioli et al. 2024). Monitoring one's own mental state relies on sensitivity to introspective information and responses to alternations in the autonomic nervous system (processes that usually do not involve conscious awareness) and, at later stages, the processing of such information in frontal cortex areas (see Fleming and Dolan 2012; Molenberghs et al. 2016; Morales et al. 2018; Paul et al. 2015 for a comprehensive review of the neural networks implicated in implicit and explicit metacognitive processing). At later stages, metacognition relies on conscious awareness of information to a greater extent than at earlier stages.

In the administered test, participants were asked to perform explicit metacognitive judgments (by assigning confidence ratings to their decisions when they had to focus their attention on consciously evaluating their own performance). Making explicit metacognitive judgments in this high‐level visuo‐spatial cognitive task required the integration of introspective and visuo‐spatial information (binding of information received via introspection and via sensory channels). Earlier studies have shown that ASD individuals sustain their attention on introspective information for a longer time (Botvinick and Cohen 1998; Schauder et al. 2015), resulting in better evaluations of their own mental states. Moreover, compared with TD individuals, ASD individuals need longer delays to connect cross‐domain and cross‐modal information (Feldman et al. 2018; Stevenson et al. 2016). Owing to longer delays, frontal areas have more time to make conscious metacognitive judgments on the basis of more accurate introspective information, leading to higher metacognitive accuracy (assigning lower confidence to the trials on which the likelihood of an error is higher). This is a potential proximate mechanism underlying enhanced metacognition in the ASD population in a mental rotation task.

Although earlier studies have shown impaired cognitive flexibility in the ASD population, especially if the instructions were not given explicitly and had to be inferred from the results of previous behavioral responses (Teunisse et al. 2001), as in the trading game we administered during the experiment, we did not observe differences in re‐learning speed (cognitive flexibility) between the ASD and TD groups. As an emergent property of executive functions, cognitive flexibility allows living beings to adjust their behavioral responses to changes in the environment, and such adjustments might need (a) engaging and disengaging certain cognitive mechanisms; or (b) reassessing input information if the regularities in the environment change but use the same set of cognitive mechanisms to generate behavioral responses. The first property can be explored by a task‐switching paradigm, and the second—by a set‐shifting paradigm. In the trading game we administered, the task remains the same throughout the experiment, and participants need to react to the rule changes (the acquired regularities allowed participants to generate behavioral responses optimizing their profit; when the rules are changed, the same response is suboptimal, and new responses—based on a new set of rules—need to be generated, yet the cognitive mechanisms underlying extraction of regularities and generation of optimal responses is the same throughout the game). We suggest that differences in cognitive flexibility can only be observed in a task‐switching paradigm, which requires switching between sets of cognitive mechanisms, i.e., engaging some mechanisms and disengaging other mechanisms.

Rohde et al. (2018) suggested that the deficit in cognitive flexibility can be attributed wither to (a) internal control; or (b) impaired metacognition; or (c) rule generation. Our empirical data did not reveal any association between metacognitive efficiency and cognitive flexibility at individual level. At the group level, we have observed that higher metacognition between the groups does not always lead to group differences in cognitive flexibility. Therefore, we suggest that the deficit in cognitive flexibility is related either to internal control of engaging and disengaging cognitive mechanism, or to issues with rule generation.

Another interesting result is the association between overconfidence and adapting a behavioral response to a new set of rules (cognitive flexibility) but not between overconfidence and the initial learning of rules (learning efficiency). A number of studies have shown that people who are overconfident in their beliefs tend to have impaired metacognition. These are usually those who keep extreme views, either political or moral, and they are less tolerant of deviations from their values and consider new arguments that contradict their beliefs, which is associated with lower cognitive flexibility (Heinzelmann and Tran 2024; Rigoli 2023; Rollwage et al. 2018). In our study, we measured metacognition, cognitive flexibility, and overconfidence, showing that overconfidence is actually associated with cognitive flexibility and not with metacognition, suggesting that cognitive flexibility is a proxy that modulates metacognition rather than overconfidence affecting metacognitive efficiency directly. A SEModelling approach is required to better understand the relations between these variables and confirm the suggestion, yet our sample size is unfortunately not sufficient for modeling, and disentangling the intricate relations between meta‐bias, meta‐efficiency and cognitive flexibility remains an objective for future studies.

Our study has several limitations that must be taken into consideration before we can infer from our results that the differences which we observe in the collected samples are held at the population level. The size and composition of the ASD sample is determined by the availability of volunteers in the participating centers. The ASD population is characterized by an unbalanced sex ratio (approximately 80% males and 20% females; Werling and Geschwind 2013), and our sample is representative of this ratio. However, some studies have shown differences between male and female cognitive profiles in ASD populations (Bölte et al. 2011; Frazier et al. 2014; Koyama et al. 2009; Lai et al. 2011; Lehnhardt et al. 2016; Nydén et al. 2000), and they differ in terms of processing speed and working memory (enhanced in ASD females compared with ASD males) and low‐level visuo‐spatial perception (enhanced in ASD males compared with females). Such differences in male and female cognitive profiles in the ASD population might affect metacognitive monitoring (e.g., ASD males take more time to process information and generate more signals that are subject to metacognitive processing, leading to enhanced metacognition). It is theoretically possible that in the ASD population, metacognition is higher in males, whereas in the TD population, there are no sex‐specific differences in metacognitive efficiency (Polyanskaya et al. 2024). Should this turn out to be true, the overall result pattern could be accounted for by the unequal (yet representative of the ASD population) ratio of males and females in our samples (the ratio was intentionally matched across TD and ASD samples by deliberately recruiting the same number of males and females in the TD sample as we had in the group of ASD volunteers). This hypothesis requires additional empirical testing. We cannot compare metacognition in males and females because we have very few females (running the analysis only with males yields the same result pattern that we have reported on mixed‐sex samples).

The second limitation pertains to the debate about the domain‐specificity or domain‐generality nature of metacognition. Neuroimaging and behavioral evidence suggest that at least some aspects of metacognition are domain‐general (Faivre et al. 2018; Carpenter et al. 2019). Moreover, certain distinct neural processes may be engaged in some but not other metacognitive tasks, suggesting dissociation between the neural bases of metacognition in different domains (Chua et al. 2009; Fleming et al. 2016; Schnyer et al. 2004). This can explain the inconsistencies across studies, with some showing impaired metacognition in individuals with ASD, others reporting no observable differences in metacognitive efficiency, and enhanced metacognition in a mental rotation task in individuals with ASD, as we observed in our study. ASD individuals may be impaired or advantaged in some metacognitive tasks but not in others. If metacognitive efficiency is domain specific, then it would be difficult to justify the expectation that metacognitive efficiency in a mental rotation task would be related to cognitive flexibility in a trading game. However, given the strong relationships among adherence to extreme political and moral beliefs, readiness to accept contradicting evidence into consideration, and metacognition in low‐level perceptual tasks, which have been reported in multiple other studies (Rigoli 2023; Rollwage et al. 2018), we believe that there is sufficient rationale to link cognitive flexibility, overconfidence, and metacognitive efficiency across domains and tasks.

In this work, we tested the hypothesis that a metacognitive deficit, which allegedly accounts for restrictive behaviors in individuals with ASD, is related to cognitive flexibility. Using a task that draws on visuo‐spatial cognition—the domain in which ASD individuals excel—we revealed higher metacognitive ability in the ASD group compared to neurotypical controls. However, no association between cognitive flexibility and metacognition was observed. Both in the control and ASD groups, cognitive flexibility was impaired by overconfidence in one's performance.

## Author Contributions

**Mikhail Ordin**: conceptualization, investigation, writing–original draft, methodology, validation, writing–review and editing, formal analysis, data curation, visualization. **Natàlia Barbarroja**: funding acquisition, project administration, data curation. **Leona Polyanskaya**: conceptualization, writing–review and editing, supervision, investigation, funding acquisition, methodology, project administration, data curation, resources. **Héctor M. Manrique**: supervision, writing–review and editing, conceptualization. **Miguel Castelo‐Branco**: conceptualization, funding acquisition, project administration, supervision, resources.

## Ethics Statement

The experimental protocol and recruitment procedures was approved by the ethical committee of the University of the Basque Country (EHU/UPV, approval record M10_2022_146MR2) for data acquisition in Spain, by the ethical committee of the Psychology Faculty of the University of Coimbra (approval number CEDI/FPCEUC:70/0PE) for neurotypically developing participants, and by the ethical committee of the Medical Faculty of the University of Coimbra (approval number CEDI/FMUC:CE‐145/202) for the autistic participants.

## Conflicts of Interest

The authors declare no conflicts of interest.

## Peer Review

The peer review history for this article is available at https://publons.com/publon/10.1002/brb3.70668

## Data Availability

Data (anonymized) is available from the first or the last author, on a reasonable request, with approval from the Coimbra Institute for Biomedical Imaging and Translational Research.
